# The complete mitochondrial genome of *Ptychidio longibarbus* (Cyprinidae) and its phylogenetic implications

**DOI:** 10.1080/23802359.2025.2602366

**Published:** 2025-12-15

**Authors:** Ming Zou, Weitao Chen

**Affiliations:** ^a^ School of Nursing and Health Management, Wuhan Donghu College, Wuhan, China; ^b^ Pearl River Fisheries Research Institute, Chinese Academy of Fishery Science, Guangzhou, China; ^c^ Experimental Station for Scientific Observation on Fishery Resources and Environment in the Middle and Lower Reaches of Pearl River, Zhaoqing, China

**Keywords:** Cypriniformes, Labeoninae, Mitogenomics, High-throughput sequencing, Endemic fish

## Abstract

The first complete mitochondrial genome of Ptychidio longibarbus is reported here. The circular genome is 16,604 bp long, containing 13 protein-coding genes, 22 tRNAs, two rRNAs, and a control region, with ∼239× coverage. All genes are located on the heavy strand except nad6 and eight tRNAs on the light strand. The mitogenome shows a typical A+T bias (57.72%). Phylogenetic analysis based on 12 concatenated PCGs places *P. longibarbus* in a well-supported clade with *P. jordani* and *P. macrops*, confirming its taxonomic position and providing new genomic resources for Labeoninae systematics and conservation.

## Introduction

**Figure 1.**
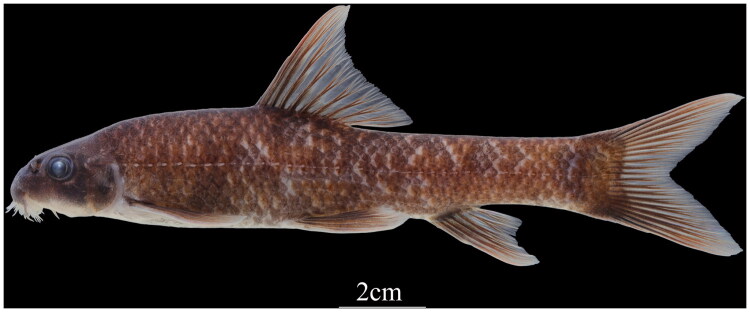
The photograph of *P. longibarbus.* Photograph by Mr. Jiahu Lan, used with permission. The specimen was collected from Guangxi province of China (108°16′N, 23°15′E), on 21 June 2023.

*Ptychidio longibarbus* Chen and Chen 1989 (Cypriniformes: Cyprinidae) is a narrowly distributed freshwater fish species endemic to China. It is known only from a few locations in the Xijiang River system, particularly from an abandoned mine shaft connected to subterranean rivers in Heshan, Guangxi (Chen and Chen 1989). This small-sized benthic species is characterized by a fusiform body with a cylindrical anterior and laterally compressed posterior, distinctive branched fleshy barbels, and a specialized subterminal mouth (Figure 1).

Despite their ecological and economic significance, the genus *Ptychidio* remains poorly studied, and the phylogenetic position of *P. longibarbus* is still uncertain due to the lack of comprehensive molecular data. Among its congeners, *P. jordani* occurs primarily in the Xijiang River system and Taiwan and is recognized as an economically important freshwater species in southern China (Wang et al. 2024), whereas *P. macrops*, distributed in the Hongshui and Mengjiang rivers of Guangxi and Guizhou, is an endangered species inhabiting clear, rocky streams. In this study, we sequenced, assembled, and annotated the complete mitochondrial genome of *P. longibarbus* for the first time. The specimen was collected from its known locality in the Xijiang River system. Our work provides essential molecular data from this poorly known species and offers a valuable reference for future taxonomic, phylogenetic, and conservation studies within the genus *Ptychidio* and related lineages in Cypriniformes.

## Materials and methods

### Sample collection

A specimen of *P. longibarbus* was collected from the upper reaches of the Disu River, Guangxi Province, China (latitude 108°16′N, longitude 23°15′E). The fish was identified based on its external morphological characteristics, and a small portion of the caudal fin was excised from the live specimen and immediately preserved in 96% ethanol for subsequent DNA extraction. The voucher specimen was deposited in the Fish Collection of the Pearl River Fisheries Research Institute, Chinese Academy of Fishery Sciences, under the accession number CXJK2023 (contact person: Weitao Chen; email: ncuskchenweitao@163.com).

### Data analysis

Total genomic DNA of *P. longibarbus* was extracted using the TIANamp Genomic DNA Kit (Tiangen, Beijing, China) according to the manufacturer’s instructions. DNA quality was assessed by 1.2% agarose gel electrophoresis, and concentration was measured using a NanoDrop 2000 spectrophotometer (Thermo Scientific, Waltham, MA). High-throughput sequencing was performed on the BGI platform with a paired-end 150 base pairs (bp) strategy.

Raw reads were quality-controlled using fastp v0.20.1 (Chen et al. 2018). The mitogenome was de novo assembled with MitoZ v3.3 (Meng et al. 2019). Filtered reads were mapped to the assembly using BWA-MEM (Li 2013), and coverage depth was calculated via SAMtools (Li H et al. 2009). Gene annotation was performed with MitoAnnotator (Iwasaki et al. 2013) and verified by BLAST against NCBI references.

For phylogenetic analysis, mitogenomes of related Labeoninae species were retrieved from NCBI. The protein-coding genes (PCGs) were identified by aligning *Danio rerio* mitochondrial proteins to each mitogenome using GeneWise v2-4-1 (Birney et al. 2004). The resulting nucleotide sequences were translated into amino acids and aligned with MAFFT v7.490 (Katoh et al. 2002). Codon alignments were generated using PAL2NAL (Suyama et al. 2006), and conserved regions were extracted with Gblocks v0.91b (Castresana 2000). The filtered PCGs were concatenated and used for phylogenetic tree construction with IQ-TREE v1.6.7 (Nguyen et al. 2015), using 1000 ultrafast bootstrap replicates. ND6 was excluded from the analysis because of its distinct evolutionary rate and base composition bias (Powell et al. 2013).

## Results

### Mitochondrial genome organization

**Figure 2.**
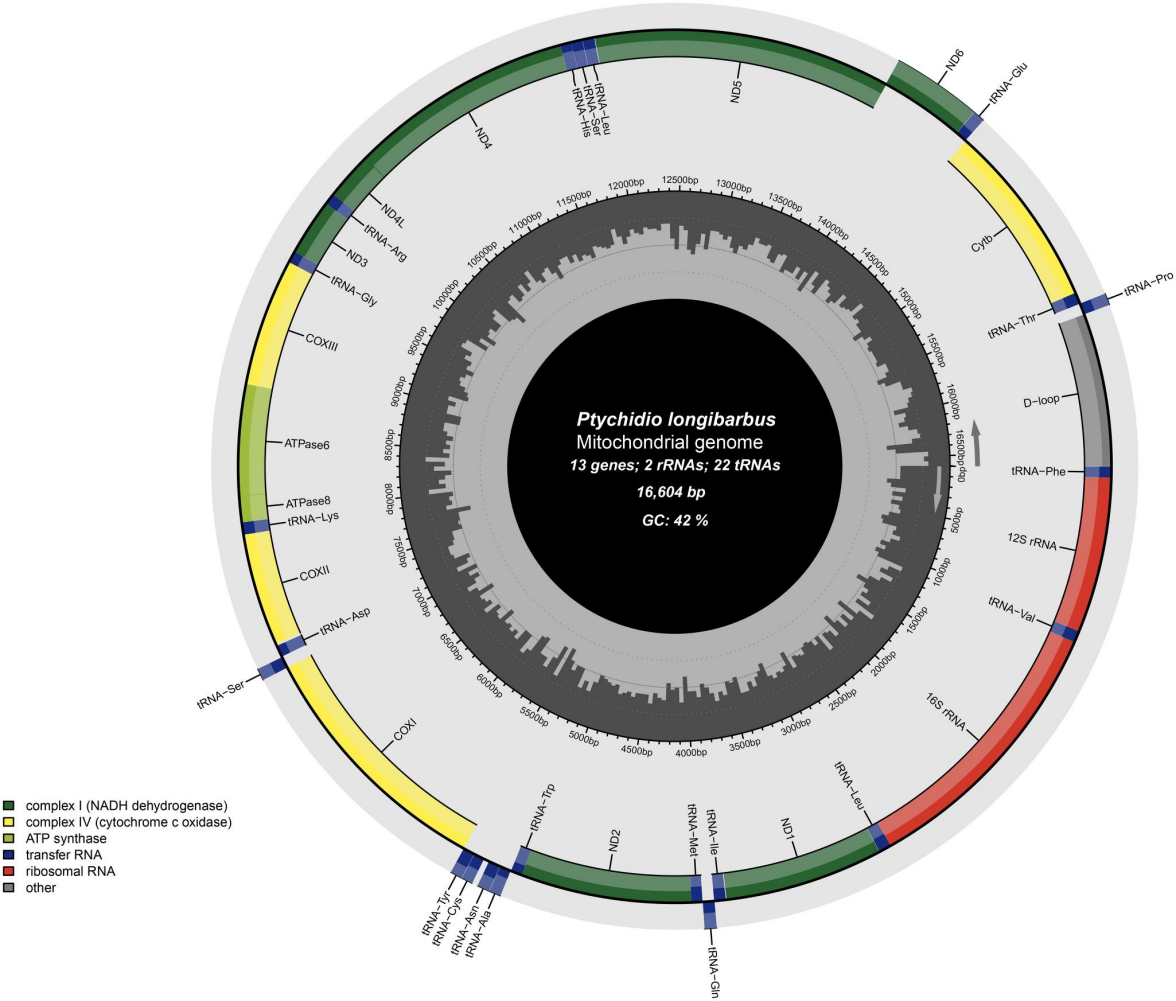
Circular feature map of *P. longibarbus* mitogenome. Genes outside the circle are encoded on the light strand and genes inside the circle are encoded on the heavy strand.

The circular mitochondrial genome of *P. longibarbus* is 16,604 bp in length, with ∼239× average coverage, and comprises 13 PCGs, 22 tRNAs, two rRNAs, and one D-loop region. (Figure 2, Supplementary Figure S1, and Table S1). Among them, most genes are encoded on the heavy (H) strand, except for *nad6* and eight tRNA genes (*trnQ*-Gln, *trnA*-Ala, *trnN*-Asn, *trnC*-Cys, *trnY*-Tyr, *trnS*-Ser, *trnE*-Glu, and *trnP*-Pro), which are located on the light (L) strand.

The nucleotide composition of the mitochondrial genome was 32.04% A, 25.68% T, 15.44% G, and 26.84% C, resulting in an overall AT content of 57.72% (Supplementary Table S2). The nucleotide composition bias between the two DNA strands was assessed by AT-skew and GC-skew, with values of 0.11 and −0.27, respectively.

The 13 PCGs range from 165 to 1824 bp in length, collectively spanning 11,409 bp. The 22 transfer RNA (tRNA) genes exhibit lengths ranging from 66 to 76 bp. A total of five overlapping regions and 15 intergenic spacers were identified across the mitogenome. The longest overlap (7 bp) occurs between *atp8* and *atp6*, and the longest intergenic spacer (33 bp) lies between *trnN-*Asn and *trnC-*Cys. Most PCGs use the typical ATG start codon, except for *cox1*, which begins with GTG. Six PCGs (*nad2*, *cox2*, *atp6*, *cox3*, *nad3*, *nad4*, and *cob*) end with incomplete stop codons (T– or TA−), while the remaining seven terminate with complete stop codons (TAA) (Supplementary Table S1). Strand asymmetry analysis of the PCGs revealed an AT-skew of 0.06 and GC-skew of −0.34 on the H-strand, while those on the L-strand showed an AT-skew of −0.52 and GC-skew of 0.50 (Supplementary Table S2).

The 12S and 16S rRNA genes are 955 bp and 1688 bp long, respectively, and are located between *trnF*–Phe and *trnL*–Leu (Figure 2 and Supplementary Table S1), with an AT content of 54.98% (Supplementary Table S2). The control region spans 939 bp, situated between *trnP*-Pro and *trnF*-Phe, and exhibits a relatively high AT content (66.24%) compared with other regions of the mitogenome, including the 13 PCGs (57.94%).

### Phylogenetic relationships

**Figure 3.**
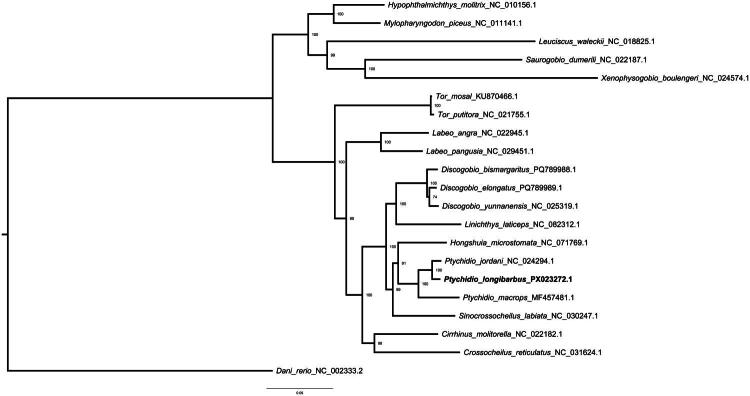
Maximum-likelihood phylogenetic tree of *P. longibarbus* and related cyprinid species inferred from concatenated nucleotide sequences of 12 mitochondrial protein-coding genes (excluding ND6). The following mitochondrial genome sequences were used: NC_010156.1 (Li SF et al. 2009), NC_011141.1 (Wang et al. 2012), NC_018825.1 (Wang et al. 2013), NC_022187.1 (Wan et al. 2015), NC_024574.1 (Tao and Zhao 2016), KU870466.1 (Sarma et al. 2022), NC_021755.1 (Sati et al. 2014), NC_022945.1 (Yang et al. 2012), NC_029451.1 (Goldfarb et al. 2025), PQ789988.1 (Cheng et al. 2025), PQ789989.1 (Cheng et al. 2025), NC_025319.1 (Goldfarb et al. 2025), NC_082312.1 (Goldfarb et al. 2025), NC_071769.1 (Goldfarb et al. 2025), NC_024294.1 (Zhao et al. 2016), PX023272, MF457481.1 (Li and Han 2018), NC_030247.1 (Goldfarb et al. 2025), NC_022182.1 (Zhang et al. 2015), and NC_031624.1 (Goldfarb et al. 2025), NC_002333.2 (Broughton et al. 2001). The tree was constructed using IQ-TREE under the best-fit substitution model with 1000 ultrafast bootstrap replicates. *P. longibarbus* (PX023272, this study) was clustered with *P. jordani* (NC_024294.1) and *P. macrops* (MF457481.1), forming a well-supported clade within the subfamily Labeoninae. Bootstrap support values ≥70% are shown at the nodes. *Danio rerio* (NC_002333.2) was used as an outgroup.

To investigate the phylogenetic position of *P. longibarbus*, we reconstructed a phylogenetic tree based on the complete mitochondrial genomes of related cyprinid species. *P. longibarbus* clustered most closely with *P. jordani* and formed a well-supported clade (bootstrap value = 100%) with *P. macrops* (Figure 3). This *Ptychidio* clade was further grouped with *Hongshuia microstomata* and *Sinocrossocheilus labiata*, forming a sister lineage to *Linichthys laticeps* and members of *Discogobio* (i.e. *D. bismargaritus*, *D. elongatus*, and *D. yunnanensis*), with strong support (bootstrap >98%). More distantly related species such as *Cirrhinus molitorella*, *Crossocheilus reticulatus*, *Labeo angra*, *L. pangusia*, and members of *Tor* and *Hypophthalmichthys* were resolved into separate clades.

## Discussion and conclusions

The complete mitochondrial genome of *P. longibarbus* exhibits compositional and structural features typical of vertebrate mitogenomes. Its gene content and organization are highly conserved compared with those of its congeners *P. jordani* and *P. macrops* (Zhao et al. 2016; Li and Han 2018). Similar to these closely related species, the mitogenome of *P. longibarbus* comprises 13 PCGs, 22 tRNA genes, two rRNA genes, and a control region, with most genes encoded on the heavy strand except for ND6 and eight tRNAs. The control region, located between the *trnP*-Pro and *trnF*-Phe, exhibits a high AT content and strong variability, which are characteristic features of mitochondrial control regions in teleosts (Cheng et al. 2025). Several PCGs terminate with incomplete stop codons, which are believed to be completed by post-transcriptional polyadenylation, ensuring proper translation termination in mitochondrial genomes (Ojala et al. 1981). In addition, the base composition shows clear strand asymmetry, likely reflecting the bias introduced by strand-asynchronous replication (Gomes-Dos-Santos et al. 2023).

Maximum-likelihood phylogenetic analysis using IQ-TREE with 100 ultrafast bootstrap replicates placed *P. longibarbus* firmly within the genus *Ptychidio*, in agreement with previous morphological classifications (Chen and Chen 1989). The robust bootstrap support further confirms its evolutionary placement and supports the utility of complete mitochondrial genomes in resolving cyprinid phylogenies.

Overall, this study provides the first complete mitogenome of *P. longibarbus*, contributing valuable genomic data for future phylogenetic and taxonomic studies within Labeoninae and broader Cyprinidae lineages.

## Supplementary Material

Supplementary Figure S1.jpg

## Data Availability

The genome sequence data supporting the findings of this study are publicly available in GenBank at NCBI (https://www.ncbi.nlm.nih.gov/) under accession number PX023272.1. The associated BioProject, SRA, and BioSample numbers are PRJNA1309007, SRR35111246, and SAMN50722647, respectively.
