# Supplementary figures and images for: The complete mitochondrial genome of Ptychidio longibarbus (Cyprinidae) and its phylogenetic implications
Source: Mitochondrial DNA B Resour. 2025 Dec 15;11(1):59–63. doi: 10.1080/23802359.2025.2602366 (PMC12707096; doi:10.1080/23802359.2025.2602366)

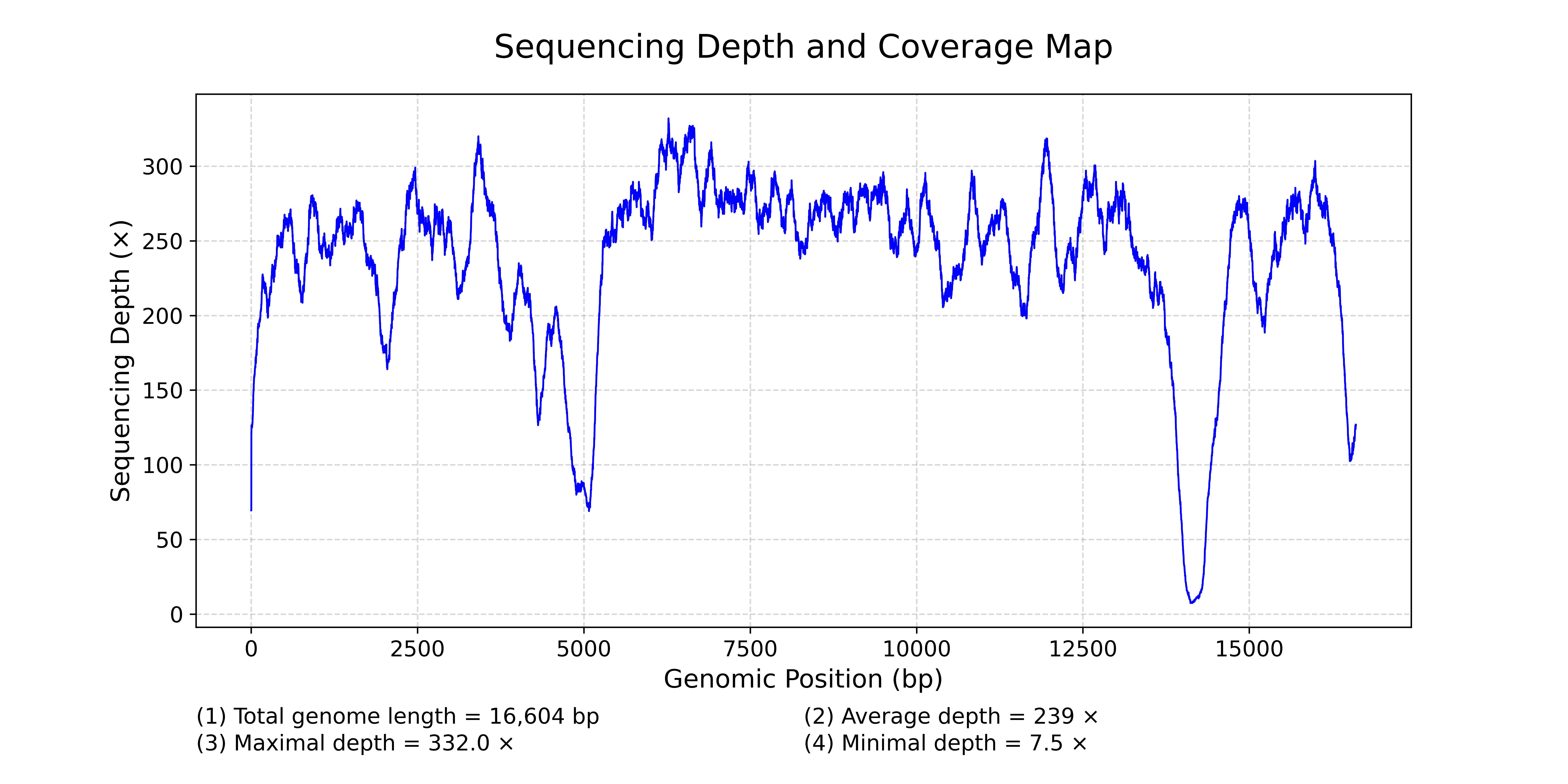

Supplement: Supplementary Figure S1.jpg [file TMDN_A_2602366_SM0187.jpg]
